# Non-tuberculous mycobacteria pulmonary disease in Texas: an analysis of 8 years of hospital discharge data

**DOI:** 10.3389/ftubr.2026.1740382

**Published:** 2026-05-29

**Authors:** Alex Baham, Jotam G. Pasipanodya, Devanshi Mehta, Susan McBride, Shashikant Srivastava

**Affiliations:** 1 Institute for Health Innovations, Data Science, and Research, The University of Texas at Tyler, Tyler, TX, United States; 2 Division of Infectious Diseases, Vanderbilt University Medical Center, Nashville, TN, United States; 3 Section of Pulmonary and Critical Care, Department of Medicine, University of Texas at Tyler School of Medicine, Tyler, TX, United States; 4 Division of Infectious Diseases, Department of Medicine, University of Texas at Tyler School of Medicine, Tyler, TX, United States; 5 Department of Cellular and Molecular Biology, University of Texas Health Science Center at Tyler, Tyler, TX, United States

**Keywords:** geographical variation, hospital discharge data, non-tuberculous mycobacteria, pulmonary disease, Texas

## Abstract

**Background:**

Non-tuberculous mycobacterial (NTM) pulmonary disease (PD) is a recognized chronic condition associated with high healthcare utilization. Texas statewide NTM diagnosis-coded inpatient discharges per 100,000 discharge records (healthcare utilization) have not been systematically studied.

**Methods:**

We analyzed Texas hospital inpatient discharge public use data between 2016 and 2023 for all 254 Texas counties. MS-EXCEL and SPSS were used for data collection and analysis.

**Results:**

Out of 23.33 million hospital discharge records, 12,587 were identified to have an NTM diagnosis-coded inpatient discharge. Among the patients with NTM, 3.3% of the patients were aged 0–17 years, 19.5% were 18–44 years, 29.5% were 45–64 years, and 47.7% were 65 + years old. The NTM diagnosis-coded inpatient discharge rate in Texas was calculated to be 59.9/100,000 records (range 46.1–62.6). A total of 815 NTM cases were reported in Northeast Texas and 4,203 cases in the Gulf Coast counties. The total number of in-hospital deaths among the NTM-coded discharges was 692 (5.5% of total NTM cases). The number of patients with NTM covered under Medicare Part A was 5,102 and 1,521 were on Medicaid.

**Conclusions:**

Based on the hospital discharge data analysis, there appears to be regional variation in the prevalence of NTM disease in Texas. Further studies are needed to determine these variations and to improve our understanding of NTM-PD in Texas.

## 1 Introduction

Non-tuberculous mycobacteria (NTM) are environmental mycobacteria; several of them are pathogenic to humans, causing “hard-to-treat” pulmonary disease (PD) (1). NTM are comprised of multispecies organisms whose risk factors (2–6) and prevalence vary significantly and are frequently associated with contaminated soil and municipal water sources (7–11). *Mycobacterium avium* complex (MAC), *Mycobacterium abscessus*, and *Mycobacterium kansasii* are among the top three NTM (1). As progress is made to improve the diagnosis and treatment of tuberculosis, resulting in fewer cases, there has been a notable increase in the reported prevalence and incidence of NTM-PD in the United States (11) for incompletely understood reasons. Challenges within the field include diagnosis, determination of who would benefit from treatment, less-than-desirable treatment success rates, and frequent recurrence even in the setting of culture conversion.

Middle-aged to elderly (>65 years of age) women of Caucasian descent without a history of smoking or obvious risk factors constitute a large proportion of patients with NTM-PD. Among the other risk groups are immunologically suppressed patients with cystic fibrosis or ciliary-related diseases, young male smokers or patients with prior tuberculosis, and patients with bronchiectasis or chronic obstructive pulmonary disease who develop rapidly progressive cavitary disease. Population prevalence studies and trends in healthcare utilization rates, either from insurance claims databases, pharmacy records, or hospital inpatient admission records, have traditionally been used to track NTM disease burden (12) and report a growing prevalence in the North American population (11–17).

The aim of this study was to estimate the yearly NTM diagnosis-coded inpatient discharges, in high-cost healthcare utilization cases, in Texas between 2016 and 2023, using Texas hospital inpatient discharge public use data (THIDPUD). Data were extracted using the International Classification of Diseases (ICD-10-CM) codes to identify the NTM cases. The American Thoracic Society (ATS)/Infectious Diseases Society of America (IDSA) pulmonary NTM disease criteria used to deﬁne the disease recommend that clinical, radiographic, and microbiological criteria are equally important, and all must be met to make a diagnosis of NTM-PD (18). Since we analyzed the hospitalization data and patients may be admitted due to other comorbidities, we refrained from classifying the identified NTM cases as “severe.” Preliminary results from this study were presented as an abstract and poster at the 2025 Colorado NTM Conference (19).

## 2 Methods

### 2.1 Rationale for the study

Since NTM-PD is not reportable, large data sets with comprehensive patient records are often hard to obtain, and diagnosis requires a complex combination of clinical symptoms paired with radiological findings and microbiological confirmation, proxy-based studies may provide an estimate of NTM disease burden in Texas.

### 2.2 Data sources and analysis

The study was approved by the University of Texas at Tyler Institutional Review Board (IRB#2024-170). We used THIDPUD collected between 2016 and 2023. We used the ICD-10-CM codes (20) A31.0 (pulmonary mycobacterial infection), A31.2 (disseminated *M. avium*-intracellulare complex, A31.8 (other mycobacterial infections), and A31.9 (mycobacterial infection, unspecified) in either the primary or secondary diagnosis fields to identify discharges associated with NTM pulmonary disease in the Texas data. We caution that since these codes were used as a proxy for NTM-PD, the data may include non-pulmonary or non-specific NTM diagnosis. Records were screened to collect data on patients with NTM as the primary or secondary diagnosis in all 254 Texas counties. A sub-analysis was performed for the 35 counties in the Northeast Texas (NETX) region, the geographic area we serve, and for 13 Texas Gulf Coast counties. Annual NTM diagnosis-coded inpatient discharge rates/100,000 discharges were estimated for each calendar year by dividing the case counts by the total number of discharges and then multiplying by 100,000. We also collected data on in-hospital deaths among NTM-coded discharges. MS-EXCEL, MS-Power BI, and SPSS were used for data collection, graphing, and analysis.

## 3 Results

### 3.1 NTM-PD in Texas

A total of 23,333,839 hospital discharge records for the years 2016–2023 were available for analysis (Table 1). Female patients accounted for 54.5% (*n* = 12,723,702) of the total hospital discharge records across all 254 Texas counties. We observed a slight decline in the number of hospitalization records in 2020, followed by a reversal of the trend (Table 1). We speculate that this observation could be due to the then ongoing COVID-19 pandemic (21). With regards to the NTM diagnosis-coded inpatient discharge records across Texas, a total of 12,587 cases were identified, out of which 5,821 (46.2%) were female. There were 2,204 (17.5%) masked gender cases, i.e., we could not assign them as male or female. Therefore, the actual number of female patients could potentially be higher or vice versa. As shown in Table 1, among the female patients with NTM-coded discharges, a large proportion (4,482, 77.0%) were Caucasian.

**Table 1:** Year-wise distribution of hospital discharge data for NTM-PD in Texas.

Year	Discharges	Female	NTM cases				
			Total	NTM rate/100K admissions	Female, *n* (%)	Caucasian female, *n* (%)	Masked^a^, *n* (%)
2016	2,837,676	1,676,511	1536	54.1	819 (53.3)	584 (71.3)	0 (0.0)
2017	2,996,158	1,642,736	1,814	60.5	825 (45.5)	632 (76.6)	398 (21.9)
2018	2,904,605	1,599,014	1,713	59.0	775 (45.2)	593 (76.5)	330 (19.3)
2019	2,905,507	1,596,139	1,818	62.6	790 (43.5)	634 (80.3)	375 (20.6)
2020	2,760,250	1,483,946	1,402	50.8	631 (45.0)	495 (78.4)	261 (18.6)
2021	2,892,602	1,551,597	1,334	46.1	632 (47.4)	491 (77.7)	243 (18.2)
2022	3,005,701	1,569,936	1,476	49.1	655 (44.4)	523 (79.8)	296 (20.1)
2023	3,031,340	1,603,823	1,494	49.3	694 (46.5)	530 (76.4)	301 (20.1)
Total	23,333,839	12,723,702	12,587	59.9 (46.1–62.6)	5,821 (46.2)	4,482 (77.0)	2,204 (17.5)
Northeast Texas (NETX)							
2016	179,628	103,791	92	51.2	50 (54.3)	46 (92.0)	0 (0.0)
2017	191,291	104,221	115	60.1	58 (50.4)	50 (86.2)	12 (10.4)
2018	186,116	101,549	103	55.3	49 (47.6)	47 (95.9)	13 (12.6)
2019	186,511	100,720	125	67.0	48 (38.4)	45 (93.8)	25 (20.0)
2020	177,022	94,060	86	48.6	31 (36.0)	31 (100.0)	14 (16.3)
2021	183,606	96,995	79	43.0	44 (55.7)	40 (90.9)	3 (3.8)
2022	182,500	94,233	123	67.4	57 (46.3)	51 (89.5)	19 (15.4)
2023	183,075	97,024	92	50.3	40 (43.5)	34 (85.0)	16 (17.4)
Total	1,469,749	792,593	815	55.5 (43.0–67.4)	377 (46.3)	344 (91.2)	102 (12.5)
Texas Gulf Coast							
2016	693,200	410,762	479	69.1	275 (57.4)	183 (66.5)	0 (0.0)
2017	711,100	393,739	587	82.5	269 (45.8)	205 (76.2)	134 (22.8)
2018	688,531	386,088	591	85.8	276 (46.7)	197 (71.4)	115 (19.5)
2019	705,613	394,638	609	86.3	252 (41.4)	181 (71.8)	112 (18.4)
2020	674,663	368,693	499	74.0	227 (45.5)	169 (74.4)	86 (17.2)
2021	715,292	387,061	453	63.3	220 (48.6)	163 (74.1)	91 (20.1)
2022	725,964	385,120	472	65.0	224 (47.5)	152 (67.9)	81 (17.2)
2023	736,124	388,147	513	69.7	243 (47.4)	160 (66.8)	123 (24.0)
Total	5,650,487	3,114,248	4,203	74.4 (63.3–86.3)	1,986 (47.3)	1,410 (71.0)	742 (17.7)

Data are shown for the 8 years between 2016 and 2023 for all 254 Texas counties and then subdivided for 35 Northeast Texas (NETX) counties and 13 Texas Gulf Coast counties. The rate is for per 100,000 (100 K) records.

a Masked means no ethnicity and gender information was available to assign the case as male or female.

Table 2 summarizes the data by age group and proportion of female patients with an associated NTM-related diagnosis. We found that across all age groups, 53.4% (range: 43.8%–70.3%) were female, with the highest proportion (70.3%) among the 18-to-44-year age group. The distribution of NTM cases by age group showed that female patients accounted for 46.7% (range: 29.3%–67.2%) and the percentage of NTM cases among elderly women was high (48.6% in the 66–74-year age group and 67.2% in the 75+ age group). However, since sex/gender was masked/unknown for 2,204/12,587 NTM-coded discharge records, the actual disease burden may be different among female patients and across age groups. Regarding the region-wide distribution, the number of reported NTM cases in NETX was 815 and 4,203 in the Gulf Coast region of Texas.

**Table 2:** Age and sex-wise NTM case distribution in Texas overall and in the Northeast Texas and Gulf counties.

Age group	Total discharges			NTM cases								
				Texas			Northeast Texas			Gulf Coast		
	*n*	Female, *n* (%)	Masked, *n* (%)	*n*	Female, n (%)	Masked, *n* (%)	*n*	Female, *n* (%)	Masked, *n* (%)	*n*	Female, *n* (%)	^a^Masked, *n* (%)
0–17	4,156,138	2,006,630 (48.3)	102,615 (2.5)	416	215 (51.7)	11 (2.6)	13	7 (53.8)	0 (0.0)	145	85 (58.6)	0 (0.0)
18–44	6,188,956	4,348,603 (70.3)	714,272 (11.5)	2,460	720 (29.3)	1,145 (46.5)	100	35 (35.0)	52 (52.0)	770	197 (25.6)	373 (48.4)
45–64	5,247,348	2,300,064 (43.8)	675,560 (12.9)	3,709	1,367 (36.9)	867 (23.4)	231	77 (33.3)	36 (15.6)	1,314	525 (40.0)	303 (23.3)
65–74	3,514,838	1,702,037 (48.4)	184,247 (5.2)	2,753	1,337 (48.6)	143 (5.2)	220	92 (41.4)	12 (5.5)	956	492 (51.5)	55 (5.8)
75+	4,213,433	2,364,011 (56.1)	66,378 (1.6)	3,249	2,182 (67.2)	38 (1.2)	251	166 (66.1)	2 (0.8)	1,018	687 (67.5)	11 (1.1)

a Masked means no gender information was available to assign the case as male or female.

We found an in-hospital death rate of 5.5% among the NTM-coded discharges between 2016 and 2023, with Table 3 reporting the distribution across different age groups and the proportion of female patients with NTM-PD. We could not find any association between NTM-PD and length of hospital stay. Among the patients who died with reported NTM-coded discharges, the mean length of hospital stay was (mean ± standard deviation) 21.0 ± 60.9 days, compared to 11.4 ± 17.6 days in NTM-coded discharges with no reported death. Regarding healthcare insurance usage, the top two insurance providers were Medicare Part “A,” with 5,102 cases, and Medicaid, with 1,521 cases.

**Table 3:** Year-wise, age group, and sex-based NTM-coded mortality records in Texas and regional variations.

Year	Texas			NETX			Gulf Coast		
	deaths	NTM cases	%	deaths	NTM cases	%	deaths	NTM cases	%
2016	100	1,536	6.51	4	92	4.35	32	479	6.68
2017	92	1,814	5.07	2	115	1.74	25	587	4.26
2018	102	1,713	5.95	8	103	7.77	29	591	4.91
2019	76	1,818	4.18	4	125	3.20	19	609	3.12
2020	66	1,402	4.71	4	86	4.65	16	499	3.21
2021	74	1,334	5.55	3	79	3.80	21	453	4.64
2022	83	1,476	5.62	6	123	4.88	24	472	5.08
2023	102	1,494	6.83	6	92	6.52	29	513	5.65
Mortality in different age groups									
0–17	5	416	1.20	0	13	0.00	0	145	0.00
18–44	121	2460	4.92	4	100	4.00	36	770	4.68
45–64	197	3,709	5.31	7	231	3.03	50	1,314	3.81
65–74	170	2,753	6.18	14	220	6.36	49	956	5.13
75+	202	3,249	6.22	12	251	4.78	60	1,018	5.89
Mortality by gender									
Male	294	4,562	6.44	15	336	4.46	79	1,475	5.36
Female	267	5,821	4.59	15	377	3.98	77	1,986	3.88
Masked/unknown	134	2,204	6.08	7	102	6.86	39	742	5.26

### 3.2 NTM-PD in NETX

The NETX region comprises 35 counties, referred to as the DSHS Health Service Region 4/5 (https://utsystem.edu/netx/) (22). The population of the NETX region is ∼1.6 million people, i.e., ∼5.4% of the Texas population (22). There were a total of 1,469,749 hospital discharge records available for the NETX region between 2016 and 2023 (Table 1). As shown in Table 1, a total of 815 NTM diagnosis-coded inpatient discharge records could be identified; 377 were female, and among these, 344 records were Caucasian female patients. The proportion of female patients with NTM-PD in the 18–44, 45–64, and 75+ age groups was calculated to be 35.0%, 33.3%, and 66.1%, respectively (Table 2). The number of in-hospital deaths in NTM-coded discharges in NETX, over the 8-year study duration, was calculated to be 37/815 (4.5%) (Table 3). The mean length of hospital stay for patients with an NTM-coded discharge record who died in NETX was 11.3 ± 10.8 days, which was not statistically different from that of those with NTM-coded discharge records who did not die (10.9 ± 16.6 days).

### 3.3 NTM-PD in the Texas Gulf Coast region

As per the 2020 Census, the Texas Gulf Coast region, which includes 13 counties, is home to ∼7.3 million (∼25% of the state's total population) people, and nearly 65% of the region's population is concentrated in Harris County (23). We identified 5,650,487 inpatient discharge records from the Gulf Coast region, and 4,203 were associated with NTM (Table 1). As shown in Table 1, 1,986/4,203 (47.3%) of the NTM cases were female, and 1,410/1,986 (71.0%) were Caucasian female patients. The proportion of female patients with NTM in the 18–44 and 45–64 age groups was calculated to be 25.6% and 67.5%, respectively (Table 2). The number of NTM-coded death records in the Gulf Coast region between 2016 and 2023 was 195/4,203 (4.6%), and 77 were female patients (Table 3). The length of hospital stay of the patients who died and had an NTM-coded diagnosis was 28.6 ± 106.9 days vs. 11.6 ± 16.4 days for the patients for whom no death was reported.

## 4 Discussion

There has been a global increase in both the number of clinical isolates and the prevalence of NTM-PD (4, 12, 15, 16, 24–26). However, an exact determination of the NTM-PD burden is challenging due to case selection and difficulties in subspecies identification, and NTM infections are not reportable. Even after a diagnosis of NTM infection is established, a significant proportion of patients do not encounter favorable treatment outcomes (27, 28). Thus, continued observation even after treatment completion is required. Without a doubt, NTM-PD imposes a significant financial burden on patients and the healthcare systems. However, estimation of the NTM-PD cost burden was not the objective of this study, and such cost-linkage studies are warranted.

A recent study by Spaulding et al. (25) reported geographic variation in the distribution of NTM in the United States, and an increasing trend in the prevalence of NTM-PD cases. Analyzing data for the period between 2009 and 2013, this study reported that MAC was the predominant NTM with 75% of all the NTM isolates and up to 61% of the NTM isolates in the West South Central region, including Texas (25). Another study by Winthrop et al (5), utilizing the statewide laboratory-based surveillance project for NTM in Oregon over 2 years, 2005–2006, highlighted the limited NTM epidemiological information available regarding the prevalence of NTM-PD in the United States. However, no such study was found that specifically highlighted the NTM disease burden in Texas.

Thus, this retrospective study analyzed Texas inpatient hospital discharge data over 8 years (2016–2023) to determine the NTM-PD burden in Texas and whether there exists geographic variation within the state to better inform policy decisions. On the state level, first, we screened >23 million hospital discharge records using ICD-10-CM codes to identify cases associated with NTM-PD. We found that, in Texas, female patients consistently accounted for more than half of the hospital discharge records associated with NTM-PD. Second, despite a large number of masked cases for race/gender/ethnicity, a large proportion (77.0%) of the female patients with NTM-PD were Caucasian. Third, similar to a previous report citing an increasing incidence rate of NTM-PD in the aged population (29), we found that, in Texas, the percentage of NTM-PD cases among women was high, with rates of 48.4% in the 65–74-year age group and 67.2% in the 75+ age group. Thus, age appears to be a significant risk factor among all others for developing NTM-PD in women. Fourth, while we could not find any association between NTM-PD and length of hospital stay, 695 deaths were reported in the NTM-coded discharge records between 2016 and 2023, and the proportion was higher in patients over 65 + years of age, i.e., 372 (53.5%).

Regarding regional differences in NTM-PD prevalence in Texas, in the NETX region, which has 35 counties, out of 1,469,749 hospital discharge records, 815 NTM-PD cases were counted. Caucasian female patients accounted for a large proportion of the female NTM-PD cases, i.e., 344/377 (91.2%) in NETX. We observed high mortality (14/220, 6.36%) in the 65–74 age group. In this study, we found that the proportion of female gender cases was higher in the Gulf counties compared to other Texas counties. The proportion of Caucasian female patients among patients with NTM-PD in the Gulf counties (71.0%) was similar to the Texas state average (77.0%), in contrast to NETX (91.2%). The distribution of patients across age groups and mortality in the NTM-coded records was not different over the 8-year study duration. Our study also provides a statewide birds-eye view of the estimate of NTM-PD burden (in terms of NTM diagnosis-coded inpatient discharges/100,000) among beneficiaries covered via different insurance plans, with Medicare Part A and Medicaid covering a large proportion of the patients by virtue of the age distribution; however, the burden was not as high as that reported in a previous Medicare-based study where the annual prevalence (cases/100,000 persons) increased ∼2.5-fold between 1997 and 2007 (30). These findings and previous reports indicate a potential need for additional healthcare support in aging patients with NTM-PD.

Despite meaningful findings on the NTM-PD burden in Texas, our hospital discharge data analysis study has several limitations. First, we used data from a hospitalized population, whereas the majority of patients with NTM-PD are diagnosed and managed in an outpatient setting. Therefore, the NTM-PD burden we report may not be the same for outpatient populations. Second, in the deidentified data, we could not identify multiple hospitalizations for any patient. Therefore, it is a possibility that a single patient could be represented more than once each year, resulting in an overestimation of the true impact of NTM-PD. Third, gender was masked for 17.5% of the total NTM-PD cases. Therefore, the proportion of female patients we report here may not be a true representation of the NTM-PD burden in Texas. Fourth, the hospital discharge data we used for these analyses did not include microbiological information to determine which NTM (e.g., MAC vs. *M. abscessus*) is the prevalent pathogen in Texas, and if the dominant species are different in NETX and the Gulf Coast region. Fifth, the analyzed data did not include the required information to perform a subanalysis to determine other pulmonary comorbidities in the identified NTM cases and what, if any, independent risk factors for NTM-PD were present.

In conclusion, despite the limitations of our data, NTM-PD is high in Texas, particularly among elderly Caucasian women in certain geographic areas. Further studies are needed to systematically define the actual prevalence of NTM disease in non-hospitalized persons to identify the risk factors for disease susceptibility as well as environmental exposure.

## Data Availability

The original contributions presented in the study are included in the article/Supplementary Material, further inquiries can be directed to the corresponding author.
